# Supplementary material for: Getting More Out of Biomedical Documents with GATE's Full Lifecycle Open Source Text Analytics
Source: PLoS Comput Biol. 2013 Feb 7;9(2):e1002854. doi: 10.1371/journal.pcbi.1002854 (PMC3567135; doi:10.1371/journal.pcbi.1002854)
Supplement: Dataset S1 — GATE software. Dataset S1 bundled with this paper contains a distribution of GATE (or see http://gate.ac.uk/download/). (TGZ) [file pcbi.1002854.s001.tgz › plos-gate/doc/releases.html]

GATE Releases


GATE Releases

## GATE Developer/Embedded 7.0 (build 4195), February 8th 2012

GATE Developer/Embedded version 7.0 is now available for download
from Sourceforge (follow the download link from
http://gate.ac.uk/download).

See the release announcement
for more details.

## GATE Developer 6.1, Mimir, Teamware, Cloud, May 6th 2011

Details of this release are available here.

## GATE 6.0 (build 3764), November 8th 2010

GATE version 6.0 is now available for download
from Sourceforge (follow the download link from
http://gate.ac.uk/download).

See the release announcement
for more details.

## GATE 6.0-beta1 (build 3686), August 21st 2010

The first beta of GATE version 6.0 is now available for download
from Sourceforge (follow the download link from
http://gate.ac.uk/download).

GATE 6.0 is largely backwards-compatible with previous versions, but
there are a few incompatible changes in the WordNet support and the
ontology-aware JAPE matching support. See the
changelog
for full details.

## GATE 5.2.1 (build 3581), May 6th 2010

GATE version 5.2.1 is now available for download from Sourceforge
(follow the download link from http://gate.ac.uk/download).

This is a bug-fix release which resolves a number of bugs reported
since the release of version 5.2. See the
changelog
for full details.

## GATE 5.2 (build 3566), Apr 28th 2010

GATE version 5.2 has been superseded by 5.2.1, which fixes a number
of important bugs.

This release concentrates on adding functionality to the GATE
Embedded API, adds utility methods for use on a JAPE RHS, and improves
support for the Groovy language in GATE, as well as fixing a number of
bugs in plugins and the GATE Developer GUI.

See the
changelog for
full details.

## GATE 5.1 (build 3431), Dec 8th 2009

GATE version 5.1 is now available for download from Sourceforge
(follow the download link from http://gate.ac.uk/download).

See the release announcement
for more details.

## GATE 5.1 beta2 (build 3402), November 4th 2009

The second beta of GATE 5.1 is now available for download (follow the download link from http://gate.ac.uk/download). This release fixes a regression introduced in the beta 1 installer which caused ANNIE to fail when run on Windows.

## GATE 5.1 beta1 (build 3397), October 30th 2009

The first beta of GATE 5.1 is now available for download (follow the download link from http://gate.ac.uk/download). The core of this release is stable but the newer features may have bugs.

## GATE 5.0 (build 3244), May 29th 2009

GATE version 5.0 is now available for download from Sourceforge (follow the download link from http://gate.ac.uk/download).

See the release announcement for more details.

## GATE 5.0 beta1 (build 3048), October 31st 2008

The first beta of GATE 5.0 is now available for download (follow the download link from http://gate.ac.uk/download). The core of this release is stable but the newer features may have bugs.

## GATE 4.0 (build 2752), July 12th 2007

GATE version 4 is now available for download from Sourceforge (follow the download link from http://gate.ac.uk/download).

Version 4 includes major new facilities for annotation indexing and search, machine learning, scaleable ontology support from OWLIM (http://www.ontotext.com/owlim/ ), ontology-based document annotation, and parallel corpus alignment. There is a raft of efficiency and infrastructure improvements including revamped HTML handling, Java 5 support and numerous optimisations and bug fixes. The set of plugins available has expanded to include around 150 components, which integrate with many high-quality language processing tools from the wider research community (http://gate.ac.uk/gate/doc/plugins.html).

GATE is free and open (under the LGPL licence) and you are very welcome to use it for both commercial and research purposes. A number of businesses now use GATE in production applications, from large corporates like Thompson to startups like Garlik or Innovantage. Research, teaching and student users represent a high proportion of labs working on language and knowledge worldwide.

Plans for the future include:

- a new version of the JAPE finite state annotation processing language
- more and better integration with related systems and tools
- SAFE, the Semantic Annotation Factory Environment: http://gate.ac.uk/safe/

See the changelog for more details.

GATE is developed on Ubuntu (and other less advanced operating systems).

## GATE 3.1 (build 2270), April 7th 2006

This is the final release of version 3.1.

New features in 3.1 (see also next section) include:

- UIMA interoperability layer, paid for by IBM's TJ Watson labs (thanks folks!)
- a revised and reworked ontology abstraction API that generalises over Sesame, JENA and KAON for language technology purposes
- OntoText's new JAPE compiler, which makes ANNIE run 200% as fast as previously and in general achieves JAPE speed-ups of 2-5 times.

...and a whole bunch of bug fixes!

There are a few backwards incompatibilities (see the changelog for more details).

## GATE 3.1 beta 1 (build 2168), December 16th 2005

This is the first release (beta 1) of version 3.1. This core of this release is stable, but some new features may have bugs, and there are some backwards incompatibilities (see the changelog for more details).

New features in 3.1 include:

- UIMA interoperability layer, paid for by IBM's TJ Watson labs (thanks folks!)
- a revised and reworked ontology abstraction API that generalises over Sesame, JENA and KAON for language technology purposes
- OntoText's new JAPE compiler, which makes ANNIE run 200% as fast as previously

...and a whole bunch of bug fixes!

## GATE 3.0 (build 1846), January 14th 2005

This is the final release of version 3.0. This is a stable release and supercedes all previous versions.

New features include:

- completely rewritten document viewer/editor
- extensive ontology support
- a new plugin management system
- lots more CREOLE plugins, including Chemistry Tagger, Montreal Transducer, RASP Parser, MiniPar, Buchart Parser, MinorThird, NP Chunker, Stemmer, TreeTagger, Probability Finder, Crawler, Google PR, SVM Light, ...
- faster JAPE compiles, and removal of dependency on Sun Java compiler
- separate .jar files and Tomcat classloading fixes
- transparent XML-based application serialisation

See chapter 2 (Change Log) of the User Guide for full details of what has changed.

**Compatibility:** for version 3 we decided that it was worthwhile making a few incompatible changes in order to clean up a couple of things that have caused trouble in previous versions. For example: some recent classes have been renamed (particularly the ontologies support classes); a few new events have been added; datastores created by version 3 will probably not read properly in version 2. If you have problems use the mailing list and we'll help you fix your code!

Version 3.1 will be a bug fix and minor adjustments to new features release; send us your comments.

GATE is free software, developed using public research funds. If you find it useful, don't keep it a secret!

Thought for the day: did you know that if Walt Disney was working now, most of his films would be illegal? Check out the Electronic Frontier Foundation, or take a look at Lessig's **Free Culture**.

## GATE 3.0 (build 1810, Release Candidate 1), December 7th 2004

Since beta 1 of version 3 we have stabilised the system, and made all pre-release final changes. This RC1 release is for testing of the installation routines and backwards compatibility analysis. Please report all bugs and errors. Version 3.0 FCS will be very early 2005.

## GATE 3.0 (build 1717, Beta 1), August 6th 2004

This release is dedicated to the memory of Jeremy Black (main instigator of the ETCSL projects).

This is the latest release (is it **not** production quality). The  user guide. The  developer documentation. The download page.

See chapter 2 (Change Log) of the User Guide for full details of what has changed.

**Highlights**

New features include:

- completely rewritten document viewer/editor
- extensive ontology support
- a new plugin management system
- separate .jar files and a Tomcat classloading fix
- lots more CREOLE components (and some more to come soon)

**Compatibility**

For version 3 we decided that it was worthwhile making a few incompatible changes in order to clean up a few things that have caused trouble in previous versions. For example: some recent classes have been renamed (particularly the ontologies support classes) and a few events added (see below); datastores created by version 3 will probably not read properly in version 2. If you have problems use the mailing list and we'll help you fix your code!

**Contributions**

A big "thank you" to the following contributors of new code to this release (please let me know if I missed you out!):

- OntoText for contributing the OntoGazetteer and various other components: http://www.ontotext.com/
- Ontos International AG for the new JAPE debugger: http://www.ontosearch.com/en/company/.
- Mark Greenwood for several CREOLE components: http://www.dcs.shef.ac.uk/~mark
- Angus Roberts for several CREOLE components: http://www.dcs.shef.ac.uk/~angus/software.html.
- Luc Plamondon for the Montreal transducer [not bundled in beta 1]: http://www.iro.umontreal.ca/~plamondl/mtltransducer/.
- Media Style for the CoNLL ANNIE wrapper.
- Osamu Masutani of Denso IT Laboratory, Inc. for many useful suggestions.

**See also**

- Melandra, whose Peter Crowther sometimes helps out on the mailing list
- David Corney's BioRAT GATE/ANNIE/JAPE-based bio-informatics extraction tool.
- Thanks to Ian Roberts for lots of hard thinking about class loaders: http://www.dcs.shef.ac.uk/~ian
- The Open University's AquaLog system
- Medwrite Inc.'s medical informatics systems

## GATE 2.2 (build 1350), July 29th 2003

The last production quality release.

Developer documentation for this release.

Download.

## GATE 2.1\_01 (build 1170), February 14th 2003

The 2.1 release.

Developer documentation for this release.

Download.

## GATE 2.0 (build 835), March 14th 2002

The full-function version of 2.0.

Developer documentation for this release.

## GATE 2.0 Beta 1 (build 721), December 3rd 2001

The first (fairly) stable full-function version of 2.0.

Developer documentation for this release.

## (Very) old releases

- gate-2.0alpha3-build516
- gate-2.0alpha2-build484
- gate-2.0alpha1-build427

| gate home |
